# The Unified Narcissism Scale–Revised: Expanding Measurement and Understanding of Narcissism Across Cultures

**DOI:** 10.1177/10731911231191435

**Published:** 2023-08-08

**Authors:** Danushika Sivanathan, Boris Bizumic, Wangtianxi Li, Junwen Chen

**Affiliations:** 1 School of Medicine and Psychology, Australian National University, Acton, ACT, Australia

**Keywords:** narcissism, cross-cultural, measurement, item response theory

## Abstract

The study of narcissism has been hindered by conceptual, theoretical, and measurement in-consistencies. In this article, we report two studies that tested a novel unified conceptualization and theoretical approach to narcissism using the Unified Narcissism Scale–Revised. Study 1 revised the recently developed Unified Narcissism Scale to construct a preliminary 40-item measure in a sample of 395 American participants (*M*_age_ = 41). We confirmed the five-factor first-order model, the two-factor second-order model, and the one-factor third-order model. Study 2 considered the cross-cultural performance of the revised scale in the Chinese language in China (*N* = 326, *M*_age_ = 25.5 years) and in the English language in Sri Lanka (*N* = 354 *M*_age_ = 28.7 years) and constructed a final 35-item measure. In conducting these studies, we have demonstrated the cross-cultural importance of entitlement and self-esteem to the conceptualization of narcissism and suggest that the negative relationship between narcissism and agreeableness may be culture-specific to Western samples (as evidenced by the absence of this relationship in non-Western samples). In this article, we have constructed a measure of narcissism that has refined our understanding of the construct and created a tool to capture this understanding.

Narcissism is a complex personality trait that has evolved in its conceptualization, theory, and measurement over the years. It is recognized as a multidimensional construct with grandiose and vulnerable dimensions (Krizan & Herlache, 2018; Miller et al., 2017). Grandiose narcissism refers to an inflated sense of self, dominance in interpersonal situations, and exhibitionism, whereas vulnerable narcissism refers to low self-esteem, hypersensitivity to criticism and negative feedback, and a sense of self contingent on social evaluation (Krizan & Herlache, 2018; Pincus et al., 2009). Miller et al. (2017), who based their conceptualization of narcissism on the five-factor model, argue that antagonism is the shared core, whereas Krizan and Herlache (2018), in their narcissism spectrum model, postulate that entitlement is the shared core.

We share the theoretical position of Krizan and Herlache (2018), where narcissism is conceptualized with entitlement as the binding feature of its subtypes. Although entitlement is a facet of antagonism, the specificity of entitlement is central to narcissism. Antagonism is a broad trait that includes multiple other facets such as, callousness, immorality, and distrust, and include both belief systems and antagonistic behaviors (Crowe et al., 2018). Entitlement, on the other hand, specifically captures the belief that one is special and deserves more than others (Campbell et al., 2004). Research has consistently shown that entitlement connects both grandiose and vulnerable narcissism (Krizan & Herlache, 2018; Krizan & Johar, 2012; Wink, 1991). Even within research exploring the five-factor model of narcissism, there is recognition that the entitlement facet is especially characteristic of narcissism (Crowe et al., 2019). Finally, recent network analyses that investigated the facets central to both grandiose and vulnerable narcissism found that entitlement, not the broad antagonism trait, was the factor to connect the two narcissism dimensions (Dinić et al., 2022).

The literature reviewed thus far has focused on narcissism at a broad trait level. Exploring the mechanisms underlying narcissism is likely to assist in delineating the centrality of entitled self-beliefs from the antagonistic behaviors and relational dynamics that are a consequence of it (Campbell et al., 2004; Crowe et al., 2018). In their model of dynamic narcissism, Edershile and Wright (2021) postulate narcissism as a complex system, where phenotypic presentations of narcissism emerge from complex underlying processes. Their empirical work found that entitlement was associated with momentary grandiosity and vulnerability (Edershile & Wright, 2021). Based on these empirical findings, they argue that entitlement is the “putative core” of narcissism and is the distal cause of the observed antagonistic behaviors. It appears that based on both trait and process models of narcissism, the specific facet of entitlement appears more central to narcissism than the broad antagonism trait. Consequently, and in line with the narcissism spectrum model (Krizan & Herlache, 2018), we conceptualized narcissism to consist of grandiose and vulnerable narcissism dimensions, with entitlement as the shared core. Therefore, any scale constructed to capture narcissism must ensure all its factors strongly relate to external measures of entitlement.

Grandiose narcissism is based on an approach orientation with behaviors used to defend and maintain an inflated sense of self (Krizan & Herlache, 2018). This sense of superiority manifests as narcissistic leadership characterized by a belief system that one knows best and is therefore competent to participate as a leader and is accompanied by a focus on vanity and being physically attractive (Krizan & Herlache, 2018; Raskin & Terry, 1988). Vulnerable narcissism, on the other hand, has an avoidant approach to sustaining an entitled low sense of self (Krizan & Herlache, 2018). To maintain a sense of self, the vulnerable narcissist has a high need for external validation and harbors fantasies of self-grandiosity. Paradoxically, this is accompanied by an elevated rejection sensitivity and shame over one’s inner world (Krizan & Herlache, 2018; Pincus et al., 2009).

Measures of narcissism often capture specialized conceptualizations or singular aspects of the construct. For example, Glover et al. (2012) constructed the Five Factor Narcissism Inventory (FFNI), initially capturing grandiose and vulnerable narcissism, and further factor approaches consisting of three subscales, namely, Agentic Extraversion (capturing unique aspects of grandiose narcissism), Neuroticism (capturing unique aspects of vulnerable narcissism), and Antagonism (capturing the shared core; Miller et al., 2015). In employing the five-factor approach, the FFNI misses nuanced elements of narcissism. For example, entitlement is only a feature of vulnerable narcissism but not across all three factors. Furthermore, the Agentic Extraversion subscale represented more typical personality with it showing positive correlations with extraversion, openness to experience, conscientiousness, negative relationship with neuroticism, and was unrelated to agreeableness (Miller et al., 2015). Finally, the factor structure of the FFNI has not been reliably replicated in cross-cultural samples (Jauk et al., 2023). Similar criticisms are made for other widely used measures such as the Narcissistic Personality Inventory (NPI; Raskin & Terry, 1988) and the Narcissistic Admiration and Rivalry Questionnaire (NARQ; Back et al., 2013), which primarily capture grandiose narcissism, and for the Pathological Narcissism Inventory (PNI; Pincus et al., 2009) and the Hypersensitive Narcissism Scale (HSNS; Hendin & Cheek, 1997), which primarily capture vulnerable narcissism.

To address the criticisms of existing measures and to reflect on the contemporary conceptualization of narcissism, Sivanathan et al. (2021) developed the Unified Narcissism Scale (UNS), which represents a unified theory and conceptualization of narcissism. UNS is a 29-item scale measuring both grandiose and vulnerable narcissism, which was constructed utilizing an item pool consisting of the items from the NPI and PNI. The UNS consists of five subscales, with two (i.e., Leadership and Vanity) measuring Grandiose Narcissism and three (i.e., Contingent Self-Esteem, Grandiose Fantasy, and Hiding One’s Needs) measuring Vulnerable Narcissism. The UNS items load strongly on the five first-order factors, which in turn load strongly on two second-order factors (i.e., Grandiose and Vulnerable Narcissism), and finally load strongly on the third-order factor of Narcissism, suggesting that at the highest level of generalization, narcissism is a unitary factor. The UNS illustrated measurement invariance between men and women, and between younger (<35) and older participants (35–80).

Despite reflecting the contemporary conceptualization at a factorial level, the UNS is not without its challenges. It does not, for example, include an entitlement factor, which might be due to entitlement being captured across all factors. However, this is yet to be demonstrated as the study did not include other measures of entitlement to show that the scale correlated strongly with entitlement. Next, items forming the Leadership subscale may not adequately capture narcissistic leadership as that factor appeared to be separate from the others (i.e., showed the weakest correlations with other factors), whereas narcissistic leadership should be substantially related to other factors of narcissism (Krizan & Herlache, 2018). This factor, among others, ought to be finessed further to adequately capture the relevant constructs. Furthermore, the scale has not been assessed for its cross-cultural validity.

Personality traits are social concepts that are crafted and expressed in cultural contexts, not despite them (Funder, 2015). As personality science widens in its conceptualizations and understanding to include non-Western cultures, there is a need for tools that have been validated in non-Western samples. Cross-culturally valid measures of narcissism can allow us to explore the universal versus culturally specific aspects of the construct. For example, research in individualist cultures has led to conceptualizations where the broad antagonism trait is at the core of narcissism. But all aspects of antagonism may not serve as useful a purpose in collectivist cultures, where group harmony is privileged above the antagonism inherent to individualistic pursuits (Singelis et al., 1995). Cultural contexts most likely impact how narcissism is expressed, including, for example, its degree of antagonism, and which facets are most relevant. This can be clarified by exploring culturally specific versus universal features of narcissism to determine which aspects of antagonism may be central to narcissism. The current study incorporates culturally diverse samples through which we hoped to construct a cross-culturally valid measure of narcissism and, through this process, explore the manifestations of narcissism in non-Western samples.

## Study 1

The primary aim of the first study was to revise the existing UNS to ensure its first-order factors more appropriately capture the manifestation and conceptualization of narcissism. As previously mentioned, the Leadership factor in the UNS showed weak correlations with the other factors alluding to it—potentially capturing healthy leadership characteristics as opposed to narcissistic leadership that is more representative of grandiose narcissism (Sivanathan et al., 2021). Based on our theorizing of what constitutes narcissism, we hypothesized that we would observe a five-order factor solution (Hypothesis 1a), with the factors Leadership and Vanity loading onto Grandiose Narcissism (Hypothesis 1b)—capturing the sense of superiority and focus on physical attractiveness characteristic of grandiose narcissism. We also hypothesized that Contingent Self-Esteem, Grandiose Fantasy, and Hiding One’s Needs would load onto Vulnerable Narcissism (Hypothesis 1c), capturing the low sense of self that requires external validation, fantasies about achievement and power, and the shame and fear around expressing one’s inner world, which are characteristic of vulnerable narcissism. We also expected a third-order factor structure of Grandiose and Vulnerable Narcissism, loading onto a common factor of Narcissism (Hypothesis 1d). Given the centrality of entitlement to narcissism, we expected that all subscales would show significant positive correlations with entitlement (Hypothesis 2).

Furthermore, as grandiose narcissism is characterized by an inflated sense of self and an approach orientation, we expected it to positively correlate with extraversion and self-esteem, and negatively with neuroticism (Hypothesis 3a). On the contrary, vulnerable narcissism is characterized by a contingent sense of self and an avoidant orientation. Accordingly, we expected it to positively relate to neuroticism and negatively to self-esteem and extraversion (Hypothesis 3b). Both subtypes of narcissism tend to be challenging in interpersonal circumstances with grandiose narcissism being characterized by interpersonal manipulativeness, and vulnerable narcissism being characterized by a sense of martyrdom and neediness in interpersonal situations (Pincus et al., 2009). Therefore, we anticipated that both grandiose and vulnerable narcissism would negatively relate to agreeableness (Hypothesis 4).

### Method

#### Item Revision

To redress the limitations of UNS, we created conceptual definitions for each factor of narcissism (see Supplemental Materials 1) and, based upon this, wrote new items for each subscale. The initial item pool sent to expert reviewers included the original 29 items from the UNS, along with 147 newly written items. Expert reviewers were identified as researchers with expertise in narcissism and an understanding of scale construction. The three reviewers rated each item in relation to the conceptual definitions as *excellent*, *fair*, or *poor*. We collated their responses and only retained new items that received a score of “excellent” from all three reviewers, along with the original 29 items. This resulted in 11 items for Contingent Self-Esteem, 15 items for Leadership, 17 items for Vanity, 14 items for Grandiose Fantasy, and 13 items for Hiding One’s Needs.

#### Participants and Procedure

The sample consisted of 414 participants from the United States who were recruited via Amazon’s Mechanical Turk. Data from 19 participants were excluded due to wishing to have their data removed, failing both attention checks in the study, and completing the survey in a very short time. The final sample consisted of 395 participants with a mean age of 41 (range = 18–78; *SD* = 13.84), and 205 participants identified as men and 187 identified as women. Participants predominantly identified as White (78.73%; see Supplemental Materials 2). The ethical aspects of the study were approved by the relevant institution and were in line with the Helsinki Declaration of Human Research.

#### Measures

##### The Unified Narcissism Scale (Sivanathan et al., 2021)

We included the UNS along with the newly written items, a total of 70 items. The items are scored on a 6-point Likert-type rating scale ranging from 1 (*not at all like me*) to 6 (*very much like me*).

##### Entitlement

We used the nine-item Psychological Entitlement Scale (PES; Campbell et al., 2004) to measure entitlement. The items were scored on a 7-point Likert-type scale ranging from 1 (*strongly disagree*) to 7 (*strongly agree*). The measure had high internal consistency in this sample (α = .93).

##### Self-Esteem

The 10-item Rosenberg’s (1979) Self-Esteem Scale was included to measure global self-esteem. It was scored on a 4-point Likert-type scale ranging from *strongly disagree* (0) to *strongly agree* (3). The measure had high internal consistency in this sample (α = .90).

##### Big Five Personality Traits

We included items from the Mini-IPIP (Donnellan et al., 2006), which captured Agreeableness (four items, α = .73), Extraversion (four items, α = .73), and Neuroticism (four items, α = .63), as these conceptually discriminate grandiose from vulnerable narcissism (Miller et al., 2011). The three subscales showed adequate internal consistency in this sample. These were scored on a 5-point Likert-type scale ranging from *strongly disagree* (1) to *strongly agree* (5).

#### Data Analysis

We ran an initial confirmatory factor analysis (CFA) based on the hypothesized factor structure to assess the item loadings using lavaan package in R (Rosseel, 2012). We conducted post hoc power analyses using semPower (Moshagen & Erdfelder, 2016) to ensure we had sufficient power to detect changes and found beta was greater than .99 for this sample. We also ran item response theory (IRT) analyses, using the R package mirt (Chalmers, 2012), to determine individual item functioning using a graded response model (GRM) due to polytomous items. Each subscale was run separately due to assumption of unidimensionality (Reise & Revicki, 2015). Sample size guidelines suggest 375 participants for 15-item scales; given our unidimensional scales had less than about 15 items each, our current sample size (*N* = 395) was sufficient to obtain stable parameters (De Ayala, 1994). Based on the CFA and IRT results, we chose eight of the best-performing items for each subscale to provide scope for further revision in cross-cultural samples. We reran CFA to confirm the hypothesized factor structures, followed by correlational analyses, to determine the correlations between the revised scale and our external validity constructs. The study and planned analyses were not pre-registered; however, all data and codes have been made publicly available at the Open Science Framework and can be accessed at: https://osf.io/kb7j9/?view_only=09e852a99fc84f3eb314ba340be6ee8a.

### Results

#### Exploratory Analyses

The IRT results showed no violations of unidimensionality, and most items met the assumption of local independence based on Yen’s *Q*3 index <|0.3| (Yen, 1984). As planned, we chose eight of the best-performing items for each of the factors based on item loadings and information curves to ensure items captured information at various points of the latent trait (see details in Supplemental Materials 3 and 4).

#### Confirmatory Analyses

We re-ran the first-order CFA using eight items per subscale to determine if the model showed adequate fit to the data. Given the sample size of 395, we chose to use alternative fit indices to determine adequate/good fit by adopting Hu and Bentler’s (1999) recommendations: adequate model fit defined as comparative fit index (CFI) > .90, standardized root mean residual (SRMR) < .08, and root mean square error of approximation (RMSEA) < .08; and strong model fit estimates defined as CFI > .95, SRMR < .06, and RMSEA < .06. Although chi-square was significant, χ^2^(730) = 1,903.7, *p* < .001, the model fitted the data acceptably based on alternative fit indices (CFI = .93; RMSEA = .06 [.06 – .07]; SRMR = .06). Therefore, Hypothesis 1a was supported where five first-order factors were observed (see the individual item loadings in Supplemental Materials 4).

We then ran the second-order factor model as hypothesized. The chi-square was significant, χ^2^(735) = 2,071.11, *p* < .001; however, most of the alternative fit indices (CFI and RMSEA) showed that the model fitted the data acceptably (CFI =.92; RMSEA = .07 [.06 – .07]; SRMR = .122 which was higher than cut-off). We found in Study 2 SRMR was acceptable. Leadership had a loading of .82 and Vanity of .86 onto Grandiose Narcissism (Hypothesis 1b), whereas Contingent Self-Esteem had a loading of .89, Grandiose Fantasy of .70, and Hiding One’s Needs of .85 onto Vulnerable Narcissism (Hypothesis 1c). The two second-order factors showed a moderate correlation (*r* = .57, *p* < .001). Finally, we also ran a third-order hierarchical model that showed identical fit as our second-order hierarchical model (Hypothesis 1d). In this structure Grandiose Narcissism loaded onto Narcissism at .80 and Vulnerable Narcissism at .71, supporting the conceptualization that grandiose and vulnerable narcissism are subtypes of the unitary construct of narcissism.

#### External Validity and Reliability

The Cronbach’s αs for all the subscales were very high (all >.90). As predicted (Hypothesis 2), the subscales all yielded very strong correlations with psychological entitlement (see Table 1). As predicted, grandiose narcissism positively correlated with extraversion and self-esteem but was unrelated to neuroticism (Hypothesis 3a). Vulnerable narcissism positively correlated with neuroticism, negatively with self-esteem, and was unrelated to extraversion (Hypothesis 3b). In line with Hypothesis 4, both grandiose and vulnerable narcissism were negatively correlated with agreeableness. The specific five subscales measuring facets of grandiose and vulnerable narcissism, in general, replicated the expected correlations for the more general subscales they belong to. Nonetheless, vanity showed a non-significant relationship with self-esteem and a positive correlation with neuroticism. Similarly, grandiose fantasy yielded a positive correlation with extraversion.

**Table 1:** Correlations Between UNS-R Total Score, Subscale Scores, and Entitlement

Variable	1	2	3	4	5	6	7	8	9	10	11	12	13
1. UNS-R Total	—												
2. GN	.89***	—											
3. VN	.86***	.51***	—										
4. CSE	.74***	.41***	.89***	—									
5. LED	.70***	.89***	.30***	.21***	—								
6. VAN	.88***	.92***	.60***	.52***	.66***	—							
7. GF	.79***	.56***	.82***	.58***	.42***	.58***	—						
8. HN	.69***	.34***	.88***	.73***	.14**	.45***	.55***	—					
9. PE	.76***	.76***	.56***	.43***	.58***	.71***	.58***	.43***	—				
10. EXT	.31***	.47***	.05	.05	.49***	.37***	.19***	−.13***	.28***	—			
11. NEU	.28***	.02	.49***	.50***	−.12*	.13**	.25***	.51***	.16***	−.25***	—		
12. AGR	−.31***	−.27***	−.28***	−.17***	−.20***	−.28***	−.19***	−.37***	−.38***	.16***	−.20***	—	
13. SE	−.22***	.11*	−.52***	−.51***	.25***	−.03	−.24***	−.60***	−.02	.33***	−.57***	.27***	—

*Note.* UNS-R = Unified Narcissism Scale–Revised; GN = Grandiose Narcissism; VN = Vulnerable Narcissism; CSE = Contingent Self-Esteem; LED = Leadership; VAN = Vanity; GF = Grandiose Fantasy; HN = Hiding One’s Needs; PE = Psychological Entitlement; EXT = Extraversion; NEU = Neuroticism; AGR = Agreeableness; SE = Self-Esteem.

* *p* < .05. **p < .01. ***p < .001.

### Discussion

In this study, we tested the integrated conceptualization of narcissism and whether the Unified Narcissism Scale–Revised (UNS-R) captures it. To do so, we first aimed to revise the item pool of the UNS to ensure that the revised scale captures the breadth of the construct. We found that the revised scale, consisting of 40 items, recreated the hypothesized first-order factor structure and the hierarchical second-order and third-order structures. We also found that the scale had excellent reliability. One of the limitations of the UNS was that there was no entitlement factor, which is the central feature of narcissism, and it was not clear how the scale related to a validated external measure of entitlement. The UNS-R subscales showed strong positive correlations with psychological entitlement, suggesting that entitlement, as theorized, strongly permeates narcissism. We anticipate that entitlement did not emerge as a separate factor as it underlies all the other factors. Therefore, separating entitlement from the individual factors would possibly capture the general manifestation of that factor as opposed to the narcissistic one. Accordingly, our conceptualization and measure clearly indicate that entitlement is so central to what narcissism is that, in contrast to other measures, it cannot be separated from any of the narcissism factors. We also replicated most of the expected external correlations for grandiose and vulnerable narcissism. Nevertheless, several correlations with external variables are worth further exploration.

First, Vanity, measuring a component of grandiose narcissism, illustrated a small positive correlation with neuroticism and showed a nonsignificant relationship with self-esteem. This is in contrary to most research that shows grandiose narcissism as a composite is negatively associated with neuroticism and positively with self-esteem (Miller et al., 2011; Raskin & Terry, 1988). Nevertheless, previous research has been critical of past scales of grandiose narcissism because they over-inflate the construct’s adaptiveness (Pincus et al., 2009). The relationship between Vanity and neuroticism, and lack of relationship with self-esteem, illustrates that grandiose narcissism as captured by the UNS-R shows a more balanced approach, capturing both adaptive and maladaptive components of grandiose narcissism. Similarly, Grandiose Fantasy demonstrated a positive relationship with extraversion, contrary to past literature that shows vulnerable narcissism is negatively or non-significantly related to extraversion (Miller et al., 2011). This finding alludes to some element of adaptive nature within vulnerable narcissism and in doing so may be capturing all aspects of vulnerable narcissism rather than only pathological aspects.

The UNS-R has shown good psychometric properties within a U.S. sample and has further elucidated the Western conceptualization of the construct. The scale in its current iteration, however, cannot be used with non-American samples without further validation. We are also unable to comment on the conceptualization of narcissism in non-Western samples. Therefore, in our next study, we aimed to further refine the scale and assess its validity in non-Western samples, and in that process discern the aspects of narcissism that are culture-specific and those that are culture-general or universal.

## Study 2

Research into narcissism and its measures have predominantly focused on Western samples (Miller et al., 2017; Pincus et al., 2009). Theorizing has broadly represented our understanding as the construct manifests in Western populations. Nonetheless, there are fundamental differences between Western and non-Western populations that are likely to impact the manifestation and understanding of a construct such as narcissism. Further, most measures of narcissism have been developed within a Western sample and show varied validity in non-Western samples (Jauk et al., 2023; You et al., 2013). In this study, we aimed to assess and revise our 40-item measure to construct one that performs robustly across U.S., Chinese, and Sri Lankan samples. In this process, we aimed to further the theoretical understanding and conceptualization of narcissism to investigate the similarities and differences between how it manifests in a Western culture (i.e., the United States, which is a vertical individualist culture; Nelson & Shavitt, 2002) and two non-Western cultures (i.e., China and Sri Lanka, which are vertical collectivist cultures; Cai et al., 2012).

Personality theory argues that personality is a relational construct. It exists within social systems, and its expression at cognitive, behavioral, and interpersonal levels is influenced and driven by the cultural context within which it exists (Funder, 2015). Theorizing on narcissism has predominantly focused on U.S. samples (Krizan & Herlache, 2018; Miller et al., 2017). The United States is a vertical individualist culture (Nelson & Shavitt, 2002), in which agency and independence are valued, along with competition over group harmony. On the contrary, Sri Lanka and China can be understood as vertical collectivist cultures, in which the self is recognized as interdependent, there is a clear authority ranking, and group harmony and obedience are highly valued (Cai et al., 2012). The socio-cultural norms of societies guide and impact individual behaviors, cognitions, and interpersonal relationships. Vertical individualist and vertical collectivist cultures share both similarities and differences that are likely to impact the manifestation and the external relationships of narcissism.

One of the differences between individualist and collectivist cultures is the focus on the self as independent versus a self that is interdependent (Singelis et al., 1995). This variation in the identification of the self is evident from a young age. Children raised in Western cultures are oriented to their own needs and uniqueness, whereas in non-Western cultures children are directed to activities to understand others’ needs and wants (Lansford, 2022). This reflects individualist cultures focusing on independence and personal values and goals, whereas in collectivist cultures the self is interdependent and one’s goals and values are often enmeshed with the groups’ goals and values (Singelis et al., 1995). Furthermore, when exploring values within societies, parenting in the United States is characterized by warmth/acceptance of the children, democratic participation, and reasoning (Lansford, 2022; Wu et al., 2002). On the contrary, parenting in China emphasizes the role of encouragement of modesty, shaming/love withdrawal as a means of promoting moral behaviors and socialization, and being directive and protective of children (Wu et al., 2002). Similarly, in Sri Lankan culture, there is a focus on the value of obedience over independence in rearing children, and there is a strong involvement of parents in children’s decision-making (Arafat et al., 2020; Ekanayake et al., 2021). These findings highlight the difference in how the self is valued within each society, with Americans valuing individual pursuits and goals, whereas the Chinese and Sri Lankans value obedience, group-based values and goals, and more collective decision-making for individual pursuits.

These values translate into what is important in a relational context. In collectivist cultures, harmony in relationships is valued over independence. In contrast, in individualist cultures, the independent self is valued over group harmony and cohesion (Singelis et al., 1995). When we consider narcissism as being entitled self-importance, to maintain this sense of self, individualists and collectivists would have different interpersonal strategies. In Western samples, narcissism has consistently been positively associated with antagonism (Miller et al., 2017), where manipulating the interpersonal contexts to receive what the self needs is a fruitful strategy. This is, however, unlikely to be as useful in a culture where the self is more intimately tied to the group. Destabilizing group harmony risks expulsion from the group, and manipulating an interpersonal context reduces the value of the group, which in turn can negatively impact one’s own self. Therefore, we would expect that both grandiose and vulnerable narcissism in Sri Lankan and Chinese samples would be positively associated with self-reported agreeableness. At least appearing as an agreeable member of a group is required to sustain that entitled self-importance (Zhou et al., 2015).

Understanding the vertical nature of these three countries helps us appreciate why we can expect entitlement to remain central to narcissism across these two cultures. Within vertical cultures, there is an acceptance of inequality among people, and the self is inherently different to others with a focus on hierarchies, authority ranking, and achievement. Indeed, research shows that there is a strong focus on need for achievement in both United States and China, with the latter being oriented toward group achievement (Salili, 1996; Ward, 1990). Given this, within vertical cultures maintaining a high sense of self is tied to achievement as it relates to various domains. All three countries of interest are characterized as “vertical” cultures. Narcissism as a construct has a strong focus on fantasies about achievement, power, a sense of entitlement, and needing to appear important (Krizan & Herlache, 2018). All of these are features that are likely to be retained and even celebrated within vertical cultures. That is why we anticipate entitlement to be the shared core of narcissism across these cultures, and why we anticipate replication of our hypothesized structure.

Based on these arguments, and arguments presented in the introduction, we formed the following hypotheses. First, we hypothesized that we would replicate a five-factor structure within Chinese and Sri Lankan samples (Hypothesis 1a) and that we would observe a hierarchical structure where Leadership and Vanity load onto Grandiose Narcissism, whereas Contingent Self-Esteem, Grandiose Fantasy, and Hiding One’s Needs would load onto Vulnerable Narcissism (Hypothesis 1b), and a third-order model where Grandiose and Vulnerable Narcissism load onto a common factor of Narcissism (Hypothesis 1c). Scalar invariance indicates that the items in the scale perform similarly across all three groups and is required to make mean-level comparisons between groups (Chen, 2007). We hypothesized that we would observe scalar invariance of our scale among the three samples from Study 1 and Study 2 given that we expect the structure of narcissism to be replicated (Hypothesis 2). We expected that grandiose and vulnerable narcissism would be positively associated with entitlement and agreeableness (Hypothesis 3a) in Chinese and Sri Lankan samples. We hypothesized that grandiose narcissism would be positively associated with extraversion and self-esteem, and negatively associated with neuroticism (Hypothesis 3b), whereas vulnerable narcissism would be positively associated with neuroticism and negatively with extraversion and self-esteem (Hypothesis 3c) in Chinese and Sri Lankan samples.

### Method

#### Participants and Procedure

##### Sample 1—Chinese Sample

Participants were recruited from the research participation platform Weidiaocha and were remunerated seven Chinese Yuan for participation. A total of 402 participants completed the survey. Responses were excluded from 49 participants who did not consent to have their data used in the analysis, and from 27 participants who failed the attention checks in the study. The final sample consisted of 326 participants with 183 participants identifying as men and 143 as women. The mean age was 25.5 years (*SD* = 6.44; range = 18–53). Except for missing information of one participant, participants’ registered residencies distributed across 29 out of the 34 provincial-level administrative divisions of China, and this matched well (*r* = .83, *p < .*001) with the proportion of population of that region in the national population of China (National Bureau of Statistics of China, 2021).

##### Sample 2—Sri Lankan Sample

Participants were recruited using various social media platforms where the study ad was published, such as Facebook, Reddit, and Instagram, with participation in the study being voluntary. The study had English fluency as an inclusion criterion where only participants fluent in English were recruited. The survey was accessed by 725 participants and completed by 401 participants. Data from 47 participants were excluded due to the not being citizens of Sri Lanka, wishing to withdraw their data, or failing attention checks. The final sample consisted of 354 participants, with 257 participants identifying as women, 93 as men, and 4 as non-binary. The mean age was 28.7 years (*SD* = 6.97; range = 18–61). The majority of the participants identified as Sinhalese (63.6%), followed by Tamil (13.8 %), and Muslim (11.3%; see Supplemental Materials 2 for the complete breakdown), which represents the population split based on ethnicity (Department of Census and Statistics, 2012).

#### Measures

##### Unified Narcissism Scale–Revised

The UNS-R scale was translated into the Chinese language using the back-translation method (Brislin, 1970). The original English version was translated into Chinese language by a bilingual psychology expert and then separately back-translated by two other bilingual psychology experts. Neither of the back-translators had prior knowledge of the English language version of the items. Subsequently, cultural and linguistic modifications were made to the Chinese language version of the scale following discussions among the translators and authors of the scale. Both the English and the Chinese versions were pilot tested with a small number of bilingual participants to ensure equivalence in responding. For the data collection, in Sri Lanka, all measures were retained in English due to the high prevalence of bilingualism in Sri Lanka, and all Sri Lankan participants were fluent in English.

##### Self-Esteem

Self-esteem was captured using the Rosenberg Self-Esteem Scale (RSES), which was retained in English for the Sri Lankan participants (Rosenberg, 1979). For the Chinese participants, we used a validated translation of the RSES (Song et al., 2011). As there is no existing validation of the RSES in Sri Lanka, we assessed its functioning using CFA. The RSES showed adequate fit in the Sri Lankan data when allowing two items’ error variances to covary (see Supplemental Materials 5 for fit indices). The measure had high internal consistency in both samples (α_CHN_ = .87; α_SL_ = .88).

##### Personality Traits

We also measured extraversion, agreeableness, and neuroticism as these are the most differentiating five-factor traits between grandiose and vulnerable narcissism. For the Chinese participants, we used a validated Chinese version of the Mini-IPIP (Li et al., 2012). We only included the items in Extraversion, Agreeableness, and Neuroticism subscales making a total of 12 items. We used the English version in the Sri Lankan sample and tested the three-factor model which showed adequate fit to the data after allowing two items’ error variances to covary (see Supplemental Materials 5 for fit indices). The internal consistencies of Extraversion (α_CHN_ = .65; α_SL_ = .75), Neuroticism (α_CHN_ = .69; α_SL_ = .60), and Agreeableness (α_CHN_ = .47; α_SL_ = .64) subscales were generally acceptable given their short length.

##### Entitlement

We measured entitlement using the nine-item PES (Campbell et al., 2004). The scale had good reliability and validity in published research using Chinese samples (Bai et al., 2020). We obtained the translation from Bai et al. (2020) and used it in our study. CFA also showed that the model based on the English version had adequate fit in the Sri Lankan sample (see Supplemental Materials 5 for fit indices). In both samples, the internal consistency was high (α_CHN_ = .85; α_SL_ = .82).

#### Data Analyses Plan

We conducted post hoc power analyses using semPower (Moshagen & Erdfelder, 2016) to ensure we had sufficient power to detect changes and found beta was >.99 for both samples. Guidelines for unidimensional IRT suggest 375 examinees for 15-item scales (De Ayala, 1994), and given that each of our subscales only included eight items, our current samples had sufficient power to achieve stable parameters. We initially ran CFA and IRT using GRM on both samples to determine item loadings, intercorrelations between factors, and individual item functioning. We used the R lavaan package (Rosseel, 2012) to run CFA and the mirt package on R to run IRT (Chalmers, 2012). Based on the output for the item loadings and item information curves on the two samples, we chose the items that performed the best across the samples to determine the final revision of the scale. We then assessed fit for the first-order, second-order, and third-order factor structures based on the revised scale. We also ran measurement invariance analyses using lavaan package on R comparing the U.S. (from Study 1), Chinese, and Sri Lankan samples. Guidelines for sample sizes for multigroup CFA are varied (Putnick & Bornstein, 2016), but we found our models were identified and we had sufficient power in our individual CFA models. Finally, to assess our other hypotheses we conducted correlational analyses.

### Results

#### Exploratory Analyses

We initially ran CFA and IRT for our first-order factor structure on the Chinese and Sri Lankan samples to determine how the items performed. For IRT we ran each factor separately due to the assumption of unidimensionality and there were no violations of unidimensionality (Reise & Revicki, 2015). Most items met the assumption of local independence based on Yen’s *Q*3 index <|0.3| across both samples (Yen, 1984). We also explored item fits in these models to determine items that showed relatively poorer fit and therefore could be dropped. We found in the Chinese sample three items, one item each for the Contingent Self-Esteem, Leadership and Hiding One’s Needs subscales, and in the Sri Lankan sample two items, one each for the Vanity and Grandiose Fantasy subscale showing weaker fit (< 0.6) and relatively flat information curves. Thus, we excluded these items that resulted in the final 35-item revised scale with seven items per subscale (see Supplemental Materials 3 for information curves), and we retained 13 items from the UNS.

#### Confirmatory Factor Analyses

We re-ran CFA in both samples to assess model fit. Given the large sample size in both samples, we chose to use alternative fit indices to determine adequate/good fit. Although chi-square was significant in both samples, χ^2^_CHN_ 550 = 1,156.22, *p* < .001; χ^2^_SL_ (550) = 1,048.05, *p* < .001, the model fitted the Chinese (CFI = .919; RMSEA = .056 [.053 – .063]; SRMR = .055) and Sri Lankan data (CFI = .925; RMSEA = .051 [.046 – .055]; SRMR = .059) acceptably based on alternative fit indices. These findings provide support for Hypothesis 1a that we would observe five first-order factors. Table 2 provides the item loadings for first-order factor structure that show that all items had strong loadings in both Chinese and Sri Lankan samples.

**Table 2:** Factor Loadings of the Final Items of the Unified Narcissism Scale–Revised for the Chinese and Sri Lankan Samples

		Factor Loading				
UNS-R Item		1	2	3	4	5
Factor 1: Contingent Self-Esteem						
CSE_1	It’s hard for me to feel good about myself unless I know other people like me.	.75/.76				
CSE_2	I am disappointed when people don’t notice me.	.74/.79				
CSE_3	When others don’t notice me, I start to feel worthless.	.82/.83				
CSE_5	I need others to acknowledge me.	.65/.68				
CSE_6	When people don’t notice me, I start to feel bad about myself.	.75/.82				
CSE_7	It’s hard to feel good about myself unless I know other people admire me.	.69/.77				
CSE_11	I spend a lot of time thinking about what other people think of me.	.69/.71				
Factor 2: Leadership						
LED_1	I see myself as a good leader.		.82/.70			
LED_2	I am a born leader.		.80/.68			
LED_7	As a leader, I know what is best for my team.		.77/.69			
LED_9	I would do a better job than any other leader out there.		.86/.70			
LED_11	If I became a leader, I would be the best.		.84/.77			
LED_12	I deserve to be the leader because I know what’s best.		.79/.75			
LED_15	I have better leadership skills than other people.		.85/.66			
Factor 3: Grandiose Fantasy						
GF_1	I often fantasize about being recognized for my accomplishments.			.79/.78		
GF_2	I often fantasize about performing heroic deeds.			.78/.80		
GF_3	I want to amount to something in the eyes of the world.			.72/.67		
GF_7	I often fantasize about being admired and respected.			.85/.78		
GF_9	I fantasize about being a hero.			.78/.81		
GF_12	In my fantasies I am a highly successful person.			.81/.67		
GF_13	I fantasize about becoming an important person in the world.			.82/.79		
Factor 4: Vanity						
VAN_6	I think others are jealous of my good looks.				.68/.57	
VAN_7	I am extremely proud of my body.				.67/.69	
VAN_9	I am proud of my good looks.				.83/.82	
VAN_11	I think I turn heads when I walk down the street.				.78/.60	
VAN_12	I am exceptionally good looking.				.74/.75	
VAN_14	I enjoy looking at myself in the mirror.				.59/.67	
VAN_17	I enjoy taking photos of myself because I look so good.				.74/.62	
Factor 5: Hiding One’s Needs						
HN_4	It’s hard to show others the weaknesses I feel inside.					.51/.56
HN_8	I would feel so ashamed if someone found out all parts of me.					.84/.78
HN_9	I become a different person when I am with others for fear of disapproval.					.79/.74
HN_10	I could never be my true self with anyone because I am too ashamed by it.					.83/.87
HN_11	I am afraid to show to others who I really am.					.82/.81
HN_12	I am often ashamed to tell anyone my real thoughts and feelings.					.80/.75
HN_13	People would avoid me if they knew who I am really deep down.					.79/.75

*Note. N*_CHN_ = 326. *N*_SL_ = 354. The order of loadings goes China/Sri Lanka. All loadings were significant at *p* < .001. UNS-R = Unified Narcissism Scale–Revised; CSE = Contingent Self-Esteem; LED = Leadership; GF = Grandiose Fantasy; VAN = Vanity; HN = Hiding One’s Needs.

We ran hierarchical CFA to assess our second-order factor structure. Like our first-order model, χ^2^ was significant in the Chinese and Sri Lankan samples, χ^2^_CHN_ (555) = 1,128.87, *p* < .001; χ^2^_SL_ (555) = 1,101.69, *p* < .001. Nevertheless, based on alternative fit indices, our model fitted the data acceptably for both the Chinese (CFI = .922; RMSEA = .056 [.052 – .061]; SRMR = .071) and the Sri Lankan (CFI = .917; RMSEA = .053 [.048 – .057]; SRMR = .080) samples providing support for Hypothesis 2a. In the Chinese sample, Grandiose and Vulnerable Narcissism were moderately correlated (*r =* .33, *p* < .001), whereas in the Sri Lankan sample they were weakly but non-significantly correlated (*r =* .16, *p* > .05).

**Figure 1:**
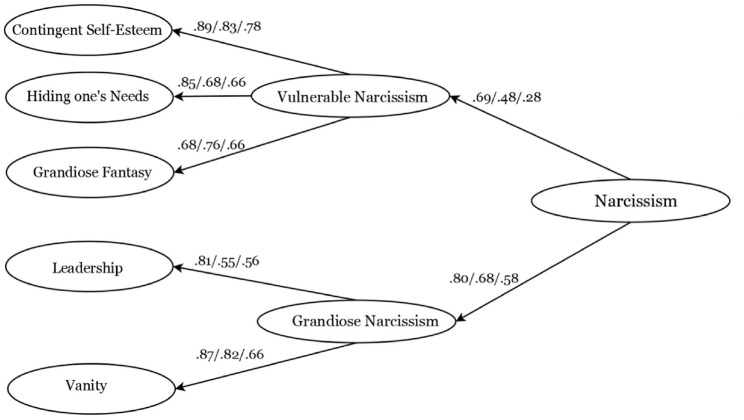
Third-Order Factor Model for UNS-R From Study 1 (the United States) and Study 2 (China and Sri Lanka) *Note. N*_US_ = 395. *N*_CHN_ = 326. *N*_SL_ = 354. The order of the loading goes the United States/China/Sri Lanka. All loadings were significant at *p* < .001.

Finally, we assessed the third-order factor structure (Hypothesis 1c). In both the Chinese and Sri Lankan samples, the fit measures indicated mathematical equivalence to the second-order model. Grandiose Narcissism loaded onto the Narcissism factor at .68 (*p* < .001) and Vulnerable Narcissism at .48 (*p* < .001) in the Chinese sample. In the Sri Lankan sample, Grandiose Narcissism loaded at .58 (*p* < .001), whereas Vulnerable Narcissism loaded slightly less than .30 at .28 (*p* < .001). The revised third-order factor model showed adequate fit in the U.S. sample, χ^2^_US_ (555) = 1535.92, *p* < .001; CFI = .931; RMSEA = .067 [.063–.071]; SRMR = .123, noting that SRMR was above cut-off; however, this seems to be the case only in the U.S. sample. Figure 1 illustrates the higher factor model, showing the loadings of the five factors onto the two second-order factors and of those onto the third-order factor.

#### Measurement Invariance

We ran multigroup CFA to assess measurement invariance across the three samples using the lavaan package for *R* (Rosseel, 2012). Measurement invariance sequentially runs increasingly restricted multigroup models in a hierarchical fashion going from an unrestricted model (configural) to restricting loadings of items to equality (metric) and then intercepts of items (scalar; Chen, 2007). Changes in CFI values <.01 and RMSEA of <.015 indicate invariance between the two models and the tested parameters being equal between groups (Chen, 2007). For purposes of cross-cultural comparison that allow mean-level comparisons, we would need at least partial scalar invariance (Steenkamp & Baumgartner, 1998). Table 3 shows the results from the multigroup CFA comparing U.S., Chinese, and Sri Lankan samples. We were able to achieve metric invariance, but when the intercepts were restricted, the model became significantly worse. Therefore, we freed 11 items’ intercepts to achieve partial scalar invariance, providing support for Hypothesis 2.

**Table 3:** Changes in Model Fit for Multigroup CFA Comparing U.S., Chinese, and Sri Lankan Samples

Model	*df*	χ^2^	Δχ^2^	*p*	CFI	ΔCFI	RMSEA	|ΔRMSEA|
Configural	1,650	3,586.54			.931		.057	
Metric	1,710	3,733.25	146.61	< .001	.928	.003	.057	.000
Scalar	1,770	4,884.57	1,151.32	< .001	.890	.038	.070	.013
Partial Scalar	1,750	4,075.95	342.70	< .001	.918	.010	.061	.004

*Note. N*_U.S._ = 395. *N*_CHN_ = 326. *N*_SL_ = 354. CFI = comparative fit index; RMSEA = root mean square error of approximation.

#### Descriptive Statistics and Reliability

Table 4 illustrates the observed means, standard deviation scores, and Cronbach’s alphas for the U.S., Chinese, and Sri Lankan samples. Across all three samples, the mean was to the center of the range with ±2*SD* in either direction. The mean scores in the U.S. sample showed more variability (higher standard deviation scores) than the Chinese or Sri Lankan samples. Cronbach’s αs for the scale and subscales were very high across all samples.

**Table 4:** Descriptive Statistics of the Unified Narcissism Scale–Revised Scale and Subscales

Scale and Subscales	United States (*N =* 395)			China (*N =* 326)			Sri Lanka (*N =* 354)		
	*M*	*SD*	α	*M*	*SD*	α	*M*	*SD*	α
UNS	3.23	1.03	.96	3.33	0.65	.93	3.14	0.66	.90
Grandiose Narcissism	3.27	1.22	.95	3.17	0.80	.91	3.14	0.80	.86
Leadership	3.60	1.24	.93	3.41	1.01	.94	3.60	0.96	.87
Vanity	2.94	1.44	.96	2.93	0.89	.88	2.68	1.01	.86
Vulnerable Narcissism	3.18	1.16	.96	3.49	0.86	.94	3.14	0.94	.92
Contingent Self-Esteem	2.93	1.38	.95	3.56	1.01	.89	2.89	1.16	.91
Grandiose Fantasy	3.59	1.32	.94	3.93	1.08	.92	3.87	1.23	.90
Hiding One’s Needs	3.03	1.35	.95	2.99	1.05	.91	2.67	1.16	.90

*Note*. Possible scores ranged from 1 to 6. UNS = Unified Narcissism Scale.

#### Correlational Analyses

Table 5 shows the correlations among narcissism and its subscales and our external validity measures. In line with Hypothesis 3a all subscales illustrated significant positive correlations with entitlement in both samples (see Table 5). In the Chinese sample only, grandiose narcissism was positively correlated with agreeableness, whereas vulnerable narcissism was unrelated to agreeableness. In the Sri Lankan sample, grandiose and vulnerable narcissism were not significantly related to agreeableness. In line with Hypothesis 3b, we found grandiose narcissism to be positively related to extraversion and self-esteem in both samples. It was negatively related to neuroticism in the Chinese sample and non-significantly in the Sri Lankan sample. Vulnerable narcissism, on the other hand, was negatively related to self-esteem, positively related to neuroticism, and unrelated to extraversion in both samples (Hypothesis 3c).

**Table 5:** Correlations Among UNS-R Subscales and External Validity Measures in the Chinese and Sri Lankan Samples

Variable	1.	2.	3.	4.	5.	6.	7.	8.	9.	10.	11.	12.
1. GN	—	.80***	.82***	.15**	.12*	.30***	−.06	.19***	−.08	−.07	.38***	.52***
2. LED	.86***	—	.31***	.17**	.13**	.31***	−.05	.15***	−.07	−.03	.30***	.49***
3. VAN	.82***	.41***	—	.09	.07	.18***	−.05	.16***	−.05	−.08	.31***	.36***
4. VN	.24***	.18**	.23***	—	.81***	.80***	.78***	−.02	.42***	.06	−.43***	.29***
5. CSE	.19***	.13*	.19***	.85***	—	.48***	.49***	.07	.46***	.09	−.38***	.24***
6. GF	.34***	.31***	.26***	.84***	.62***	—	.39***	.07	.27***	.09	−.18***	.33***
7. HN	.06	.01	.11*	.80***	.53***	.46***	—	−.19***	.30***	−.03	−.48***	.12***
8. EXT	.36***	.32***	.28***	−.07	−.02	.10	−.26***	—	−.02	.31***	.21***	.15**
9. NEU	−.26***	−.24***	−.13*	.45***	.41***	.29***	.43***	−.28***	—	.12*	−.46***	.07
10. AGR	.20***	.24***	.08	.04	.09	.16	−.14*	.28***	−.14*	—	.04	−.14**
11. SE	.36***	.33***	.27***	−.36***	−.29***	−.16**	−.45***	.38***	−.61***	.25***	—	.16**
12. PE	.40***	.38***	.28***	.45***	.36***	.52***	.24***	.11*	.16**	.08	−.06	—

*Note. N*_CHN_ = 326. *N*_SL_ = 354. Coefficients above the diagonal are from Sri Lankan sample. GN = Grandiose Narcissism; VN = Vulnerable Narcissism; CSE = Contingent Self-Esteem; LED = Leadership; VAN = Vanity; GF = Grandiose Fantasy; HN = Hiding One’s Needs; EXT = Extraversion; NEU = Neuroticism; AGR = Agreeableness; SE = Self-Esteem; PE = Psychological Entitlement.

* *p* < .05. **p < .01. ***p < .001.

### Discussion

In this study, we aimed to validate and construct a cross-culturally valid measure of narcissism and in the process explore manifestations of narcissism across cultures. Based on the data collected from China and Sri Lanka, the 40-item measure was revised to 35 items. The 35-item UNS-R showed an acceptable fit in both samples for the first-order, second-order, and third-order structures. Furthermore, measurement invariance analyses illustrated that the scale had partial scalar invariance across U.S., Chinese, and Sri Lankan samples. These findings suggest that any observed differences between these samples are due to differences in the manifestation of the construct within cross-cultural samples rather than due to measurement differences (Steenkamp & Baumgartner, 1998).

In this study, we replicated many of the expected external correlations based on previous literature supporting the external validity of the UNS-R. We found in Chinese and Sri Lankan samples grandiose narcissism was positively associated with entitlement, extraversion, and self-esteem in line with previous literature (Krizan & Herlache, 2018; Miller et al., 2011, 2017; You et al., 2013). Similarly, we found in Chinese and Sri Lankan samples vulnerable narcissism was positively associated with entitlement and neuroticism and negatively associated with self-esteem (Krizan & Herlache, 2018; Miller et al., 2011, 2017). These findings collectively allude to the validity of the UNS-R as a cross-cultural measure of narcissism.

There were some external correlations that are worth considering more carefully. Based on the theory around individualism and collectivism, we expected that narcissism would be positively related to agreeableness in both Chinese and Sri Lankan samples opposite to how narcissism manifests in Western samples. Contrary to our expectations, we only found a significant positive relationship between grandiose narcissism and agreeableness in the Chinese sample, whereas it was unrelated to grandiose narcissism in the Sri Lankan sample and to vulnerable narcissism in both samples. These findings indicate that the facets of agreeableness captured by the measure used, that is, empathy and interest in others, seem to be unrelated to narcissism in more vertical collectivist cultures. However, these findings should be treated as preliminary as we only included one measure of agreeableness, and this measure had not been previously validated in Sri Lankan samples. In this vein, all our findings about the cross-cultural structure of narcissism require further validation.

We also found in the Sri Lankan sample neuroticism was unrelated to grandiose narcissism contrary to previous research using Western samples where grandiose narcissism has been negatively related to neuroticism (Miller et al., 2011). This suggests that in a Sri Lankan context either neuroticism manifests differently in relation to other cultures or grandiose narcissism does not include this adaptive manifestation of being negatively related to neuroticism. In both Sri Lankan and Chinese samples, extraversion was unrelated to vulnerable narcissism. This adds to the existing literature that extraversion may not be as relevant to the manifestation of vulnerable narcissism, given previous studies have shown mixed results, with some studies showing a negative relationship between extraversion and vulnerable narcissism and others a non-significant relationship (Miller et al., 2011).

The current study is the first validation of UNS-R cross-culturally. Future research should explore the nomological network of narcissism within these and other cultures to better understand the aspects of the construct that are culture-specific and those that are culture-general. A major limitation of this study was that many of the external validity measures used had not been previously validated in these non-Western samples. We found that for most measures, the performance in the Chinese and Sri Lankan samples was adequate; however, the Agreeableness scale showed relatively poor performance in both the Sri Lankan and the Chinese samples. Past research has shown that Agreeableness manifests differently in China (Cheung et al., 2001), and so future research should explore the relationship between narcissism and agreeableness using the indigenous Chinese conceptualization of the construct to determine if these correlations can be replicated. As a result, we consider the findings relating to antagonism to be preliminary. Another limitation of the research conducted in Sri Lanka was that it was conducted in English, and therefore, the sample is limited to those who are fluent in English in Sri Lanka, which usually represents a middle-class, educated, and urban population. This limits the generalizability of the findings.

## General Discussion

In this paper, we report two studies that revised and further validated the UNS, creating the UNS-R, in three multicultural samples. In doing so, we aimed to construct a cross-culturally valid measure of narcissism and, in the process, explore the cross-cultural manifestation of the construct. Across cultures, we have found narcissism to consist of two dimensions, grandiose and vulnerable, that are represented by five factors. We found good support for grandiose and vulnerable narcissism being dimensions of a common narcissism factor where we replicated this third-order factor model in all samples. This indicates that both grandiose and vulnerable narcissism share a central core but deviate in how this core relates to other features of the construct. Across these two studies, we confirmed the centrality of entitlement to narcissism by using cross-cultural samples that manifested strong correlations with entitlement. In doing so, we have added to the existing literature that supports entitlement as being the central feature of narcissism (Dinić et al., 2022; Edershile & Wright, 2021; Krizan & Herlache, 2018). It is important to note, however, that in all three studies we focused on “vertical” cultures. The structuring of these cultural contexts lends itself to entitlement being a central feature of narcissism. To strongly propose the universal nature of entitlement being central to narcissism we will need to explore its manifestations in horizontal cultures, such as Scandinavian countries. We also note that there was no Entitlement factor, and therefore, future researchers interested in exploring the commonality of narcissism can do so by using an overall score on the measure.

Reflecting existing literature, we replicated the positive correlations between grandiose narcissism, self-esteem, and extraversion; negative correlations between vulnerable narcissism and self-esteem; and positive correlations between vulnerable narcissism and neuroticism (Krizan & Herlache, 2018; Miller et al., 2011; Pincus et al., 2009). Entitlement appears to combine with high self-esteem and extraversion to manifest features of grandiose narcissism such as superiority, vanity, and inflated sense of self, whereas with vulnerable narcissism entitlement combines with low self-esteem and neuroticism to manifest its features of contingent sense of self, grandiose fantasies, and shame about one’s needs. This adds to the existing literature, including the five-factor model of narcissism and the narcissism spectrum model where we could speculate that the approach orientation of grandiose narcissism is captured by extraversion and the avoidant orientation of vulnerable narcissism is captured by neuroticism (Krizan & Herlache, 2018; Miller et al., 2011, 2015).

Conversely, our cross-cultural samples did not manifest the expected positive correlations with some facets of agreeableness, suggesting that specific facets of antagonism are culturally-invariant (i.e., entitlement) while others are more culturally specific (i.e., empathy and interest in others) to narcissism. We do interpret these findings with caution, as we found our measure of agreeableness had the poorest performance in the non-Western samples. Further research into collectivist cultures must be conducted to understand the cultural specificity of antagonistic manifestations of narcissism. It is likely that not all antagonistic facets are as central, given the importance of group belonging and harmony within collectivist cultures (Singelis et al., 1995).

### Limitations and Suggestions for Further Research

Our findings related to participants from only three countries. In addition, all three samples were collected using online platforms (two from research platforms and one using social media). All three samples, therefore, likely represent urban and educated populations, and so we are unable to generalize beyond that specific cross-section for each country. Moreover, Sri Lanka and China are the only two countries that represent the broad construct of vertical collectivism. To further improve the utility of the UNS-R and our understanding of narcissism more cross-cultural research is required. Future research should aim to develop a more nuanced understanding of narcissism to illustrate the specific elements of its cross-cultural manifestation. Accordingly, future research should use representative samples from various countries around the world to further understand the aspects of narcissism that are universal and those that are culture-specific. Future research should also aim to refine our conceptualization of narcissism based on cross-cultural research, and we put forth our measure as a good starting point. As mentioned earlier, our three cross-cultural samples also represent only “vertical” cultures, and it would be useful to explore how entitlement manifests in a horizontal society that inherently devalues hierarchies (Singelis et al., 1995). Furthermore, our analyses were limited to those participants who identified as a man or woman and so we are unable to generalize our findings to gender diverse individuals.

A limitation of the UNS-R is that we are unable to comment on its utility compared to other existing measures of narcissism in this paper. In addition, the UNS-R was only partially scalar invariant requiring 11 items’ intercepts to be freed to achieve this. Group mean comparisons made using the scale may not be as reliable given the number of the item intercepts that had to be freed, with the literature having varied guidelines on what is a reasonable number of item intercepts be freed (Putnick & Bornstein, 2016). Therefore, future research should explore a shortened version of the measure that is fully scalar invariant which would allow for more reliable comparisons. The incremental validity of the UNS-R in relation to the FFNI, NARQ, and the HSNS and a prototype shortened version of the UNS-R that is scalar invariant across cultures is, however, investigated in detail in Sivanathan et al. (2023). The study included a number of personality, behavioral, and psychopathology external variables to ascertain how well the UNS-R replicated these external relationships. The study further explored whether the UNS-R was able to account for more unique variance in these relationships in comparison to existing measures of narcissism, and these relationships were explored in sample of 309 Australian residents. In their study, they found the UNS-R grandiose narcissism subscale to show superior performance to other existing measures of grandiose narcissism in both strength and coherence of external relationships, and the vulnerable narcissism subscale of the UNS-R to show similar performance to existing subscales, providing support for the incremental validity of the UNS-R. The preprint of the manuscript can be found here: https://osf.io/k6pzq/?view_only=93676f26fb5048cea72df1d11c505cd2. Furthermore, the scale is constructed containing only positively-keyed items, like the PNI, and so is vulnerable to acquiescence bias. We recommend that future research use other measures to assess for acquiescence more carefully.

The current article reports on cross-sectional studies that utilized self-report measures to explore the external validity of the UNS-R. We are unable to comment on its predictive validity, test retest reliability, or how it relates to behavioral and informant measures. Future research should conduct longitudinal studies to explore the predictive validity and test re-test reliability of the measure and consider the inclusion of behavioral assessment tools and informant measures to expand the nomological network of narcissism as measured by the UNS-R.

### Summary and Conclusion

We constructed a 35-item measure of grandiose and vulnerable narcissism that shows excellent performance in three different cultures. We replicated our first-order five-factor model, second-order two-factor model, and third-order one-factor model. We demonstrated grandiose and vulnerable narcissism are dimensions of a common narcissism construct, which share a core of entitlement. Our research further supported the centrality of entitlement to cross-cultural manifestations of narcissism. We have also shown that high self-esteem and extraversion account for the unique manifestations of grandiose narcissism, whereas low self-esteem and neuroticism capture the unique aspects of vulnerable narcissism. Holding a mirror to narcissism allowed us to develop a conceptual understanding and the tools used to measure it so that when future researchers look at narcissism, they, too, will like what they see.

## Supplemental Material

sj-docx-1-asm-10.1177_10731911231191435 – Supplemental material for The Unified Narcissism Scale–Revised: Expanding Measurement and Understanding of Narcissism Across Cultures Supplemental material, sj-docx-1-asm-10.1177_10731911231191435 for The Unified Narcissism Scale–Revised: Expanding Measurement and Understanding of Narcissism Across Cultures by Danushika Sivanathan, Boris Bizumic, Wangtianxi Li and Junwen Chen in Assessment
