# Supplementary material for: The Unified Narcissism Scale–Revised: Expanding Measurement and Understanding of Narcissism Across Cultures
Source: Assessment. 2023 Aug 8;31(4):839–54. doi: 10.1177/10731911231191435 (PMC11092293; doi:10.1177/10731911231191435)
Supplement: sj-docx-1-asm-10.1177_10731911231191435 – Supplemental material for The Unified Narcissism Scale–Revised: Expanding Measurement and Understanding of Narcissism Across Cultures [file sj-docx-1-asm-10.1177_10731911231191435.docx]

**The Unified Narcissism Scale-Revised: Expanding Measurement and Understanding of Narcissism Across Cultures**

**Supplemental Materials**

**Supplemental Materials 1: Conceptual Definitions**

**Table 1.1**

*Conceptual Definitions of the Five Factors of the First-Order Model*

| Factor | Definition |
| --- | --- |
| Contingent Self-Esteem | A reliance on others’ approval and validation to maintain a positive self-image. |
| Leadership | A tendency to believe that oneself is the best leader and knows what is best for the group. |
| Vanity | A tendency towards excessive pride and admiration about one’s physical appearance. |
| Grandiose Fantasy | A tendency to fantasize about personal success, power, ideal love, and big accomplishments. |
| Hiding One’s Needs | A tendency to hide aspects of one's self and one's needs due to feelings of shame and fear of rejection. |

**Supplemental Materials 2: Demographic Information**

**Table 2.1**

*Country of Origin and Ethnicities of Participants in the US Sample (Study 1)*

| Country of Origin | Frequency | Ethnicity | Frequency |
| --- | --- | --- | --- |
| USA | 373 (94.43%) | White/Anglo American | 222 (56.20%) |
| Canada | 10 (2.53%) | White/Anglo Canadian | 9 (2.28%) |
| UK | 12 (3.04%) | White English | 80 (20.25%) |
|  |  | Black | 26 (6.58%) |
|  |  | Hispanic American | 38 (9.62%) |
|  |  | Asian American | 6 (1.52%) |

*Note*. *N* = 395.

**Table 2.2**

*Ethnicities of Participants in the Sri Lankan Sample (Study 2)*

| Ethnicity | Frequency |
| --- | --- |
| Sinhalese | 225 (63.55%) |
| Tamil | 49 (13.84%) |
| Muslim | 42 (11.86%) |
| Burgher | 16 (4.52%) |
| Moors | 12 (3.39%) |
| Other | 10 (2.83%) |

*Note*. *N* = 354

**Supplemental Materials 3: Item Response Theory Curves**

**Figure 3.1**


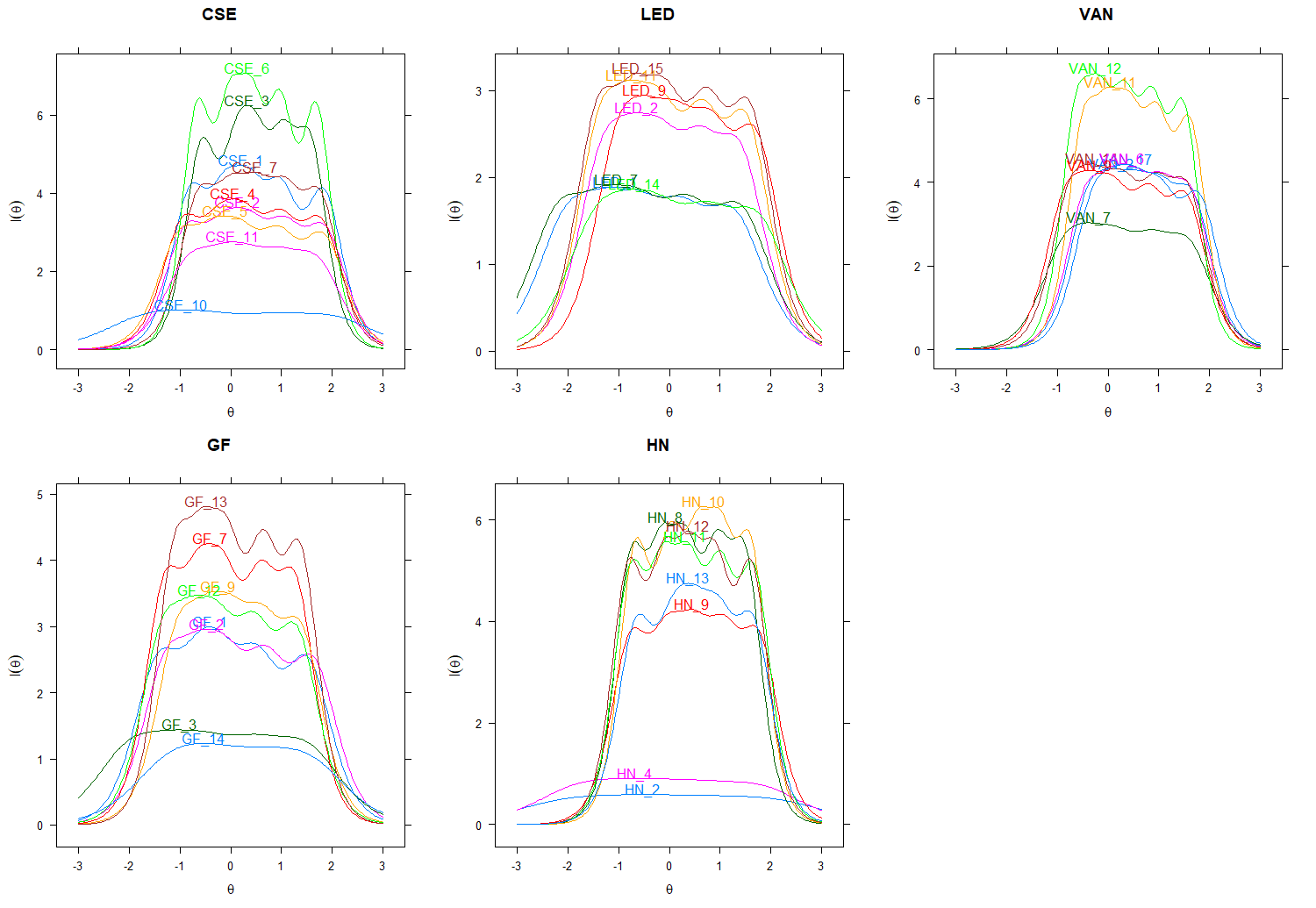
*Item Information Curves for the Subscales in Unified Narcissism Scale-Revised in the U.S. Sample*

*Note.* The figure illustrates the item information curves for each of the items within each factor. Curves that have higher peaks allude to that item providing more information than flatter curves. The graphs are truncated with θ going from -3 to +3.

**Figure 3.2**

*Test Information and Standard Error Curves for Each Narcissism Factor in the US Sample*


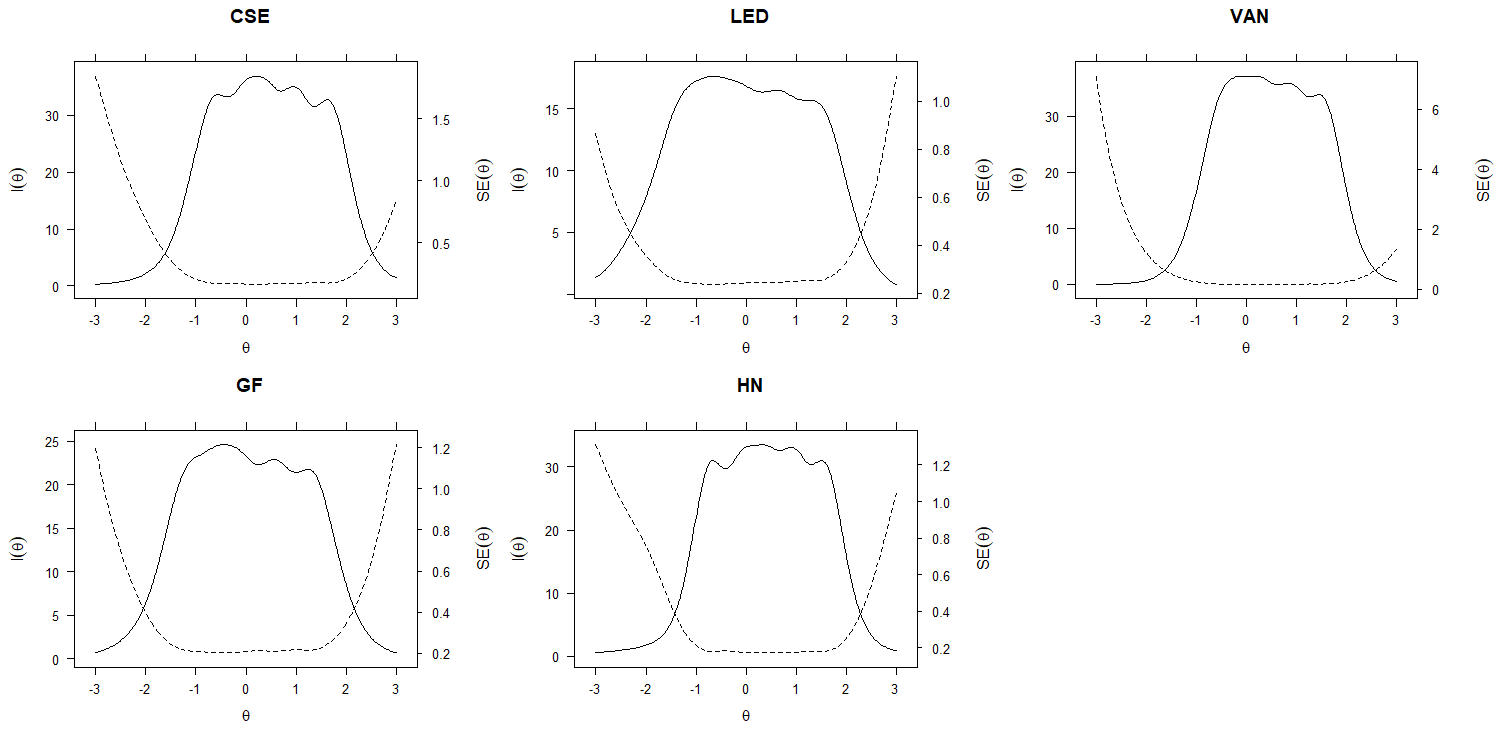


*Note.* The figure illustrates the test information curves for each of the factors. The standard error is illustrated by the dotted line.

**Figure 3.3**

*Item Information Curves for All 40 Items From the Unified Narcissism Scale in the Chinese Sample*


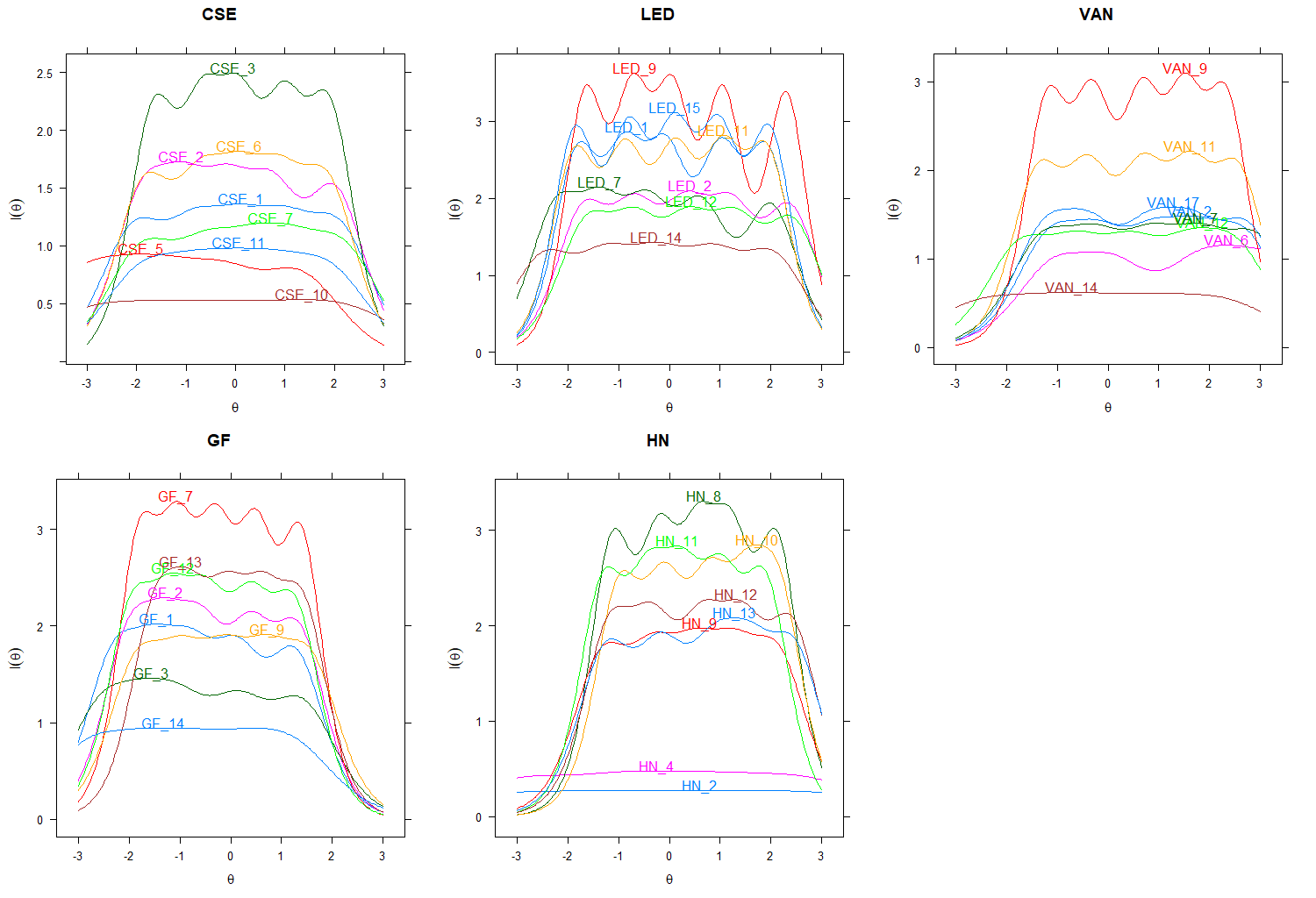


*Note.* The figure illustrates the item information curves for each of the items within each factor. Curves that have higher peaks allude to that item providing more information than flatter curves. The graphs are truncated with θ going from -3 to +3.

**Figure 3.4**

*Item Information Curves for All 40 Items From the Unified Narcissism Scale in the Sri Lankan Sample*

*
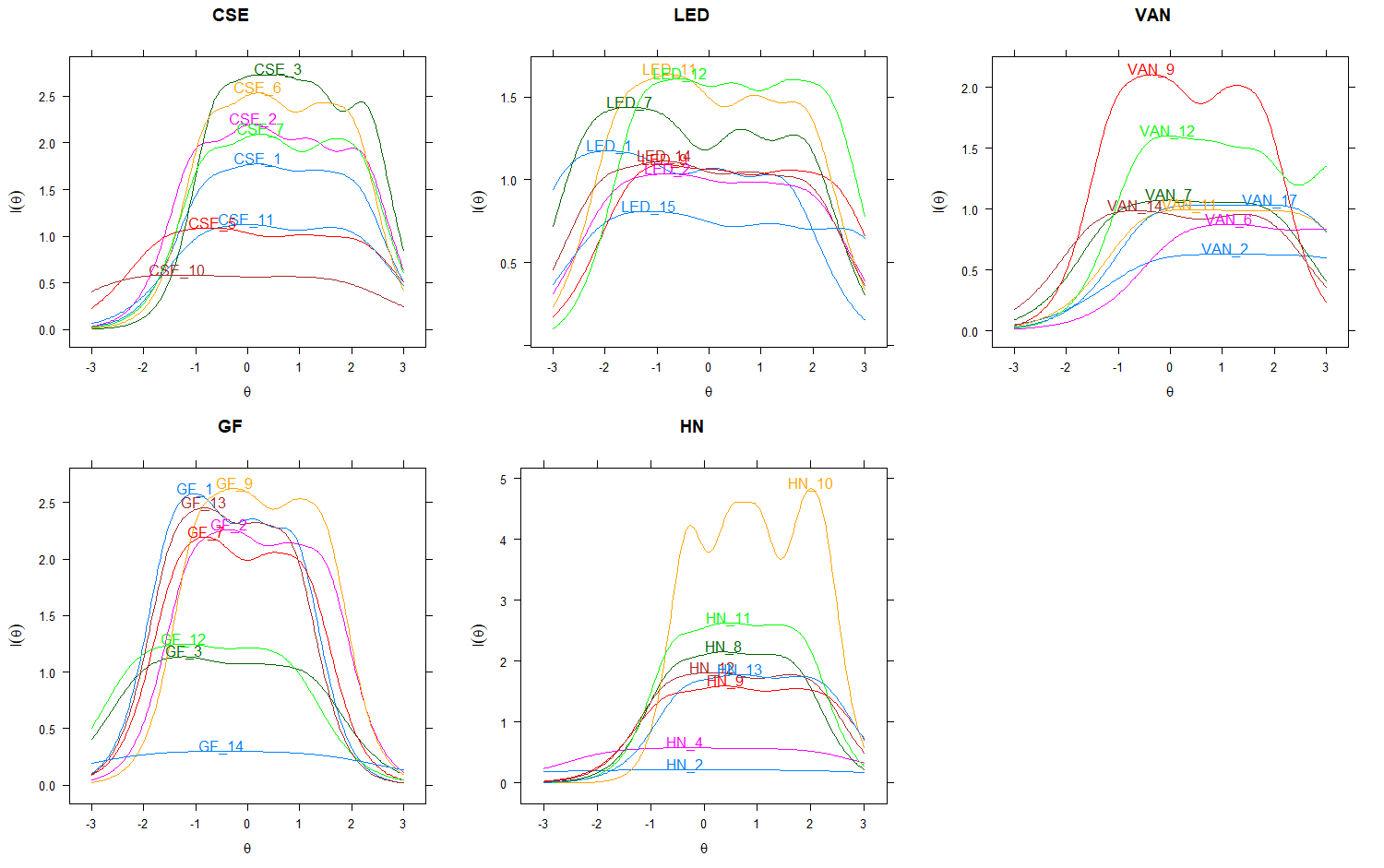
*

*Note.* The figure illustrates the item information curves for each of the items within each factor. Curves that have higher peaks allude to that item providing more information than flatter curves. The graphs are truncated with θ going from -3 to +3.

**Supplemental Materials 4: Factor Loadings**

**Table 4.1**

*Factor Loadings for All the Items of Unified Narcissism Scale Prior to Revision (US Sample)*

| UNS-R Item | | Factor Loading | | | | |
| --- | --- | --- | --- | --- | --- | --- |
|  |  | 1 | 2 | 3 | 4 | 5 |
| Factor 1: Contingent Self-Esteem | |  |  |  |  |  |
| CSE_1 | It's hard for me to feel good about myself unless I know other people like me. | .89 |  |  |  |  |
| CSE_2 | I am disappointed when people don't notice me. | .85 |  |  |  |  |
| CSE_3 | When others don't notice me, I start to feel worthless. | .89 |  |  |  |  |
| CSE_4 | When others don't respond to me the way I would like them to, it is hard for me to still feel okay with myself. | .85 |  |  |  |  |
| CSE_5 | I need others to acknowledge me. | .84 |  |  |  |  |
| CSE_6 | When people don't notice me, I start to feel bad about myself. | .90 |  |  |  |  |
| CSE_7 | It's hard to feel good about myself unless I know other people admire me. | .88 |  |  |  |  |
| CSE_8 | I need others to give me compliments to feel good about myself. | .85 |  |  |  |  |
| CSE_9 | I can’t feel good about myself if I know someone doesn’t like me. | .83 |  |  |  |  |
| CSE_10 | I feel my best when people compliment me. | .67 |  |  |  |  |
| CSE_11 | I spend a lot of time thinking about what other people think of me. | .85 |  |  |  |  |
| Factor 2: Leadership | |  |  |  |  |  |
| LED_1 | I see myself as a good leader. |  | .72 |  |  |  |
| LED_2 | I am a born leader. |  | .78 |  |  |  |
| LED_3 | I am assertive. |  | .70 |  |  |  |
| LED_4 | I insist upon getting the respect that is due me. |  | .67 |  |  |  |
| LED_5 | I can make anybody believe anything I want them to. |  | .73 |  |  |  |
| LED_6 | I don’t think my subordinates have much to contribute to improve my leadership. |  | .60 |  |  |  |
| LED_7 | As a leader, I know what is best for my team. |  | .74 |  |  |  |
| LED_8 | Whoever has me as their leader is lucky to have me. |  | .81 |  |  |  |
| LED_9 | I would do a better job than any other leader out there. |  | .85 |  |  |  |
| LED_10 | The best thing my team can do is just follow me without question. |  | .72 |  |  |  |
| LED_11 | If I became a leader, I would be the best. |  | .83 |  |  |  |
| LED_12 | I deserve to be the leader because I know what's best. |  | .88 |  |  |  |
| LED_13 | If my followers criticise me, it is because they are ungrateful, not because of anything I did. |  | .68 |  |  |  |
| LED_14 | When I lead a team, I always know what's best for all of us. |  | .80 |  |  |  |
| LED_15 | I have better leadership skills than other people. |  | .84 |  |  |  |
| LED_1 | I see myself as a good leader. |  | .72 |  |  |  |
| Factor 3: Grandiose Fantasy | |  |  |  |  |  |
| GF_1 | I often fantasize about being recognized for my accomplishments. |  |  | .84 |  |  |
| GF_2 | I often fantasize about performing heroic deeds. |  |  | .84 |  |  |
| GF_3 | I want to amount to something in the eyes of the world. |  |  | .73 |  |  |
| GF_4 | I often fantasize about having a huge impact on the world around me. |  |  | .82 |  |  |
| GF_5 | I often fantasize about accomplishing things that are probably beyond my means. |  |  | .79 |  |  |
| GF_6 | I often fantasize about being rewarded for my efforts. |  |  | .83 |  |  |
| GF_7 | I often fantasize about being admired and respected. |  |  | .88 |  |  |
|  |  |  |  |  |  |  |
|  | UNS-R Item | Factor Loading | | | | |
|  |  | 1 | 2 | 3 | 4 | 5 |
| GF_8 | I spend a lot of time dreaming about my future success. |  |  | .84 |  |  |
| GF_9 | I fantasize about being a hero. |  |  | .85 |  |  |
| GF_10 | I fantasize about other people being in awe of me. |  |  | .81 |  |  |
| GF_11 | Sometimes I can't stop myself from fantasizing about the successes I want to have. |  |  | .80 |  |  |
| GF_12 | In my fantasies I am a highly success person. |  |  | .85 |  |  |
| GF_13 | I fantasize about becoming an important person in the world. |  |  | .88 |  |  |
| GF_14 | I daydream about finding perfect love. |  |  | .69 |  |  |
| Factor 4: Vanity | |  |  |  |  |  |
| VAN_1 | I like to look at my body. |  |  |  | .87 |  |
| VAN_2 | I like to display my body. |  |  |  | .88 |  |
| VAN_3 | I like to start new fads and fashions. |  |  |  | .84 |  |
| VAN_4 | I would do almost anything on a dare. |  |  |  | .77 |  |
| VAN_5 | I am apt to show off if I get the chance. |  |  |  | .82 |  |
| VAN_6 | I think others are jealous of my good looks. |  |  |  | .87 |  |
| VAN_7 | I am extremely proud of my body. |  |  |  | .84 |  |
| VAN_8 | My good looks are one of my best qualities. |  |  |  | .87 |  |
| VAN_9 | I am proud of my good looks. |  |  |  | .86 |  |
| VAN_10 | If someone doesn't compliment my looks it is because they are jealous. |  |  |  | .80 |  |
| VAN_11 | I think I turn heads when I walk down the street. |  |  |  | .89 |  |
| VAN_12 | I am exceptionally good looking. |  |  |  | .89 |  |
| VAN_13 | I am better looking than most people. |  |  |  | .01 |  |
| VAN_14 | I enjoy looking at myself in the mirror. |  |  |  | .86 |  |
| VAN_15 | I enjoy looking at photos of myself. |  |  |  | .86 |  |
| VAN_16 | Whenever I have a chance, I like to show off my body. |  |  |  | .84 |  |
| VAN_17 | I enjoy taking photos of myself because I look so good. |  |  |  | .87 |  |
| Factor 5: Hiding Needs | |  |  |  |  |  |
| HN_1 | I hate asking for help. |  |  |  |  | .52 |
| HN_2 | I can't stand relying on other people because it makes me feel weak. |  |  |  |  | .62 |
| HN_3 | I often hide my needs for fear that others will see me as needy and dependent. |  |  |  |  | .71 |
| HN_4 | It's hard to show others the weaknesses I feel inside. |  |  |  |  | .69 |
| HN_5 | Sometimes I avoid people because I'm concerned that they'll disappoint me. |  |  |  |  | .70 |
| HN_6 | I can't tolerate the idea of others knowing about my insecurities. |  |  |  |  | .72 |
| HN_7 | I only show the world parts of me that are perfect. |  |  |  |  | .73 |
| HN_8 | I would feel so ashamed if someone found out all parts of me. |  |  |  |  | .89 |
| HN_9 | I become a different person when I am with others for fear of disapproval. |  |  |  |  | .87 |
| HN_10 | I could never be my true self with anyone because I am too ashamed by it. |  |  |  |  | .89 |
| HN_11 | I am afraid to show to others who I really am. |  |  |  |  | .88 |
| HN_12 | I am often ashamed to tell anyone my real thoughts and feelings. |  |  |  |  | .87 |
| HN_13 | People would avoid me if they knew who I am really deep down. |  |  |  |  | .85 |

*Note*. *N* = 395. Standardized loadings shown. All loadings were statistically significant at *p* < .001

**Table 4.2**

*Factor Loadings of the Revised Items of the Unified Narcissism Scale (U.S. Sample)*

| UNS-R Item | | Factor Loading | | | | |
| --- | --- | --- | --- | --- | --- | --- |
|  |  | 1 | 2 | 3 | 4 | 5 |
| Factor 1: Contingent Self-Esteem | |  |  |  |  |  |
| CSE_1 | It's hard for me to feel good about myself unless I know other people like me. | .88 |  |  |  |  |
| CSE_2 | I am disappointed when people don't notice me. | .86 |  |  |  |  |
| CSE_3 | When others don't notice me, I start to feel worthless. | .90 |  |  |  |  |
| CSE_5 | I need others to acknowledge me. | .84 |  |  |  |  |
| CSE_6 | When people don't notice me, I start to feel bad about myself. | .91 |  |  |  |  |
| CSE_7 | It's hard to feel good about myself unless I know other people admire me. | .88 |  |  |  |  |
| CSE_10 | I feel my best when people compliment me. | .65 |  |  |  |  |
| CSE_11 | I spend a lot of time thinking about what other people think of me. | .83 |  |  |  |  |
| Factor 2: Leadership | |  |  |  |  |  |
| LED_1 | I see myself as a good leader. |  | .75 |  |  |  |
| LED_2 | I am a born leader. |  | .80 |  |  |  |
| LED_7 | As a leader, I know what is best for my team. |  | .76 |  |  |  |
| LED_9 | I would do a better job than any other leader out there. |  | .85 |  |  |  |
| LED_11 | If I became a leader, I would be the best. |  | .85 |  |  |  |
| LED_12 | I deserve to be the leader because I know what's best. |  | .86 |  |  |  |
| LED_14 | When I lead a team, I always know what's best for all of us. |  | .80 |  |  |  |
| LED_15 | I have better leadership skills than other people. |  | .85 |  |  |  |
| Factor 3: Grandiose Fantasy | |  |  |  |  |  |
| GF_1 | I often fantasize about being recognized for my accomplishments. |  |  | .83 |  |  |
| GF_2 | I often fantasize about performing heroic deeds. |  |  | .84 |  |  |
| GF_3 | I want to amount to something in the eyes of the world. |  |  | .72 |  |  |
| GF_7 | I often fantasize about being admired and respected. |  |  | .88 |  |  |
| GF_9 | I fantasize about being a hero. |  |  | .86 |  |  |
| GF_12 | In my fantasies I am a highly successful person. |  |  | .84 |  |  |
| GF_13 | I fantasize about becoming an important person in the world. |  |  | .89 |  |  |
| GF_14 | I daydream about finding perfect love. |  |  | .69 |  |  |
| Factor 4: Vanity | |  |  |  |  |  |
| VAN_2 | I like to display my body. |  |  |  | .87 |  |
| VAN_6 | I think others are jealous of my good looks. |  |  |  | .86 |  |
| VAN_7 | I am extremely proud of my body. |  |  |  | .83 |  |
| VAN_9 | I am proud of my good looks. |  |  |  | .86 |  |
| VAN_11 | I think I turn heads when I walk down the street. |  |  |  | .90 |  |
| VAN_12 | I am exceptionally good looking. |  |  |  | .91 |  |
| VAN_14 | I enjoy looking at myself in the mirror. |  |  |  | .88 |  |
| VAN_17 | I enjoy taking photos of myself because I look so good. |  |  |  | .86 |  |
| Factor 5: Hiding Needs | |  |  |  |  |  |
| HN_2 | I can't stand relying on other people because it makes me feel weak. |  |  |  |  | .56 |
| HN_4 | It's hard to show others the weaknesses I feel inside. |  |  |  |  | .64 |
| HN_8 | I would feel so ashamed if someone found out all parts of me. |  |  |  |  | .89 |
| HN_9 | I become a different person when I am with others for fear of disapproval. |  |  |  |  | .87 |
|  | UNS-R Item | Factor Loading | | | | |
|  |  | 1 | 2 | 3 | 4 | 5 |
| HN_10 | I could never be my true self with anyone because I am too ashamed by it. |  |  |  |  | .91 |
| HN_11 | I am afraid to show to others who I really am. |  |  |  |  | .90 |
| HN_12 | I am often ashamed to tell anyone my real thoughts and feelings. |  |  |  |  | .89 |
| HN_13 | People would avoid me if they knew who I am really deep down. |  |  |  |  | .86 |

*Note*. *N* = 395. Standardized loadings shown. Standardized loadings shown. All loadings were statistically significant at *p* < .001

**Supplemental Materials 5: External Measures in Sri Lankan Sample**

It is important to note that the measures used to capture the external validity variables have not been psychometrically validated in a Sri Lankan sample. We ran individual CFAs to determine how the scales/subscales performed in this sample. Below are the fit indices and item loadings for each measure. Given the sample size of 354, we chose to use alternative fit indices to determine adequate/good fit (Green et al., 1997). We adopted widely utilized recommendations for acceptable alternative model fit estimates (Hu & Bentler, 1999): Comparative Fit Index (CFI) > .90, Standardized Root Mean Residual (SRMR) < .08, and Root Mean Square Error of Approximation (RMSEA) < .08; and strong model fit estimates defined as CFI > .95, SRMR < .06, and RMSEA < .06 (Hooper et al., 2008).

**Big Five Personality Subscales**

We ran a three-factor CFA to determine model fit for Extraversion, Neuroticism, and Agreeableness subscales of the mini-IPIP. The initial model showed poor fit based on chi-square and alternative fit indices (χ^2^ (51) = 238.72, *p* < .001; CFI = .792; TLI = .730; RMSEA = .102; SRMR = .086). When we allowed the error variances for the two reverse-scored items of the Agreeableness subscale (items “I Am not interested in other people’s problems” and “I Am not really interested in others”) to covary, the chi-square results were still significant (χ^2^ (50) = 124.17, *p* < .001), but based on alternative fit indices we found the model fit was acceptable (CFI = .918; TLI = .891; RMSEA = .065; SRMR = .072). It is important to note that two reverse-scored items from the Agreeableness subscale and one from the Neuroticism subscale showed somewhat lower, though statistically significant, loadings (< .30) in this sample (as shown in Table S7). Any conclusions drawn about inter-relationships with the Agreeableness subscale will need to be treated with caution.

**Table 5.1**

*Factor Loadings of the Big Five Subscales in the Sri Lankan Sample*

| Mini-IPIP Items | | Factor Loading | | |
| --- | --- | --- | --- | --- |
|  |  | 1 | 2 | 3 |
| Factor 1: Extraversion | |  |  |  |
| 1 | I am the life of the party. | .63 |  |  |
| 6 | I don’t talk a lot. (R) | .71 |  |  |
| 11 | I talk to a lot of different people at parties. | .68 |  |  |
| 16 | I keep in the background. (R) | .63 |  |  |
| Factor 2: Agreeableness | |  |  |  |
| 2 | I sympathize with others’ feelings |  | .79 |  |
| 7 | I am not interested in other people’s problems. (R) |  | .22 |  |
| 12 | I feel others’ emotions. |  | .80 |  |
| 17 | I am not really interested in others. (R) |  | .26 |  |
| Factor 3: Neuroticism | |  |  |  |
| 4 | I have frequent mood swings. |  |  | .72 |
| 9 | I am relaxed most of the time. (R) |  |  | .51 |
| 14 | I get upset easily. |  |  | .68 |
| 19 | I seldom feel blue. (R) |  |  | .18 |

*Note*. *N* = 354. Standardized loadings shown. (R) are reverse-scored items. All item loadings were significant at *p* < .001, excluding item 19 which was significant at *p* < .01.

**Rosenberg Self-Esteem Scale**

Similar to the initial fit of the Big Five personality subscales, the one-factor model of the Rosenberg Self-Esteem Scale (RSES) showed poor fit initially (χ^2^ (35) = 198.48, *p* < .001; CFI = .891; TLI = .860; RMSEA = .115; SRMR = .065); however, when two error variances were allowed to covary (items “At times I think I am no good at all” and “I certainly feel useless at times”) the fit was acceptable (χ^2^ (34) = 160.02, *p* < .001; CFI = .916; TLI = .889; RMSEA = .102; SRMR = .061). All the items showed good fit in this sample (Table S8).

**Table 5.2**

*Factor Loadings of Items in the Rosenberg Self-Esteem Scale in the Sri Lankan Sample*

| Mini-IPIP Items | | Factor Loading |
| --- | --- | --- |
| Factor 1: Self-Esteem | |  |
| 1 | On the whole, I am satisfied with myself. | .72 |
| 2 | At times I think I am no good at all. (R) | .69 |
| 3 | I feel that I have a number of good qualities. | .50 |
| 4 | I am able to do things as well as most other people. | .53 |
| 5 | I feel I do not have much to be proud of. (R) | .70 |
| 6 | I certainly feel useless at times. (R) | .67 |
| 7 | I feel that I'm a person of worth, at least on an equal plane with others. | .66 |
| 8 | I wish I could have more respect for myself. (R) | .54 |
| 9 | All in all, I am inclined to feel that I am a failure. (R) | .75 |
| 10 | I take a positive attitude toward myself. | .80 |

*Note*. *N* = 354. Standardized loadings shown. (R) are reverse-scored items. All item loadings were significant at *p* < .001.

**Psychological Entitlement Scale**

The one-factor model of the Psychologically Entitlement Scale showed acceptable model fit in the sample (χ^2^ (27) = 69.31, *p* < .001; CFI = .947; TLI = .930; RMSEA = .067; SRMR = .043) and its items had strong and statistically significant loadings (there was one reverse-scored item with a lower loading, but it was also statistically significant, *p* < .001). The item loadings can be found in Table S9 below.

**Table 5.3**

*Factor Loadings of Items in the Psychological Entitlement Scale in the Sri Lankan Sample*

| PES Items | | Factor Loading |
| --- | --- | --- |
| Factor 1: Entitlement | |  |
| 1 | I honestly feel I’m just more deserving than others. | .66 |
| 2 | Great things should come to me. | .57 |
| 3 | If I were on the Titanic, I would deserve to be on the first lifeboat! | .52 |
| 4 | I demand the best because I’m worth it. | .64 |
| 5 | I do not necessarily deserve special treatment. (R) | .27 |
| 6 | I deserve more things in my life. | .69 |
| 7 | People like me deserve an extra break now and then. | .60 |
| 8 | Things should go my way. | .59 |
| 9 | I feel entitled to more of everything. | .71 |

*Note*. *N* = 354. Standardized loadings shown. (R) are reverse-scored items. All item loadings were significant at *p* < .001.

**Supplemental Materials 6: Exploratory Analyses**

When we confirmed the second-order model in the Sri Lankan sample, we observed non-significant correlations between Grandiose and Vulnerable Narcissism factors and in the third-order model there was a weak loading of vulnerable narcissism onto the common narcissism factor. We explored this finding further by stratifying the sample by men and women and exploring the strength of correlations between grandiose and vulnerable narcissism (Table F1). Prior to doing this we needed to ensure the scale functioned similarly between men and women, and so we explored measurement invariance. We achieved strict invariance of the scale between men and women prior to this comparison, results of which are shown in Table F2. As can be observed, since ΔCFI < |.01| at each stage we can state that the measure is scalar invariant between men and women across all three samples.

Across all samples the correlation between grandiose and vulnerable narcissism was weaker in women than men. In the Sri Lankan sample, the correlation between grandiose and vulnerable narcissism amongst women was non-significant, whereas it was significant amongst men. To better assess the differences observed in the U.S. and Chinese samples in the strength of correlations we followed Zou's approach to computing confidence intervals (2007) using the R package cocor (Diedenhofen & Musch, 2015). We found that there was a significant difference in correlation between men and women in the U.S. sample but not in the Chinese sample. These findings suggest it is worthwhile in future research to more closely explore how the strength of relationship between grandiose and vulnerable may vary as a function of gender, instead only focusing on mean level differences.

**Table 6.1**

*Correlations Between Grandiose and Vulnerable Narcissism Comparing Men and Women*

|  | Sri Lanka | | China | | United States | |
| --- | --- | --- | --- | --- | --- | --- |
|  | *r* | *n* | *r* | *n* | *r* | *n* |
| Men | .28^**^ | 93 | .28^***^ | 183 | .57^***^ | 205 |
| Women | .10 | 257 | .18^*^ | 143 | .36^***^ | 187 |
| Difference | - | | .10 [-.10, .31] | | .21^*^ [.06, .37] | |

*Note.* Statistical difference analyses were not run for the Sri Lankan sample as the correlation in women was non-significant (Statistics Solutions, 2022).

**p* < .05. ***p* < .01. ****p* < .001.

**Table 6.2**

*Measurement Invariance Analyses Between Men and Women in the US, Sri Lankan, and Chinese Samples*

| Model | *df* | χ^2^ | Δχ^2^ | *p* | CFI | ΔCFI | RMSEA | ΔRMSEA |
| --- | --- | --- | --- | --- | --- | --- | --- | --- |
| US (*n*_men_ = 205; *n*_women_ = 187) | | | | | | | | |
| Configural | 1100 | 2122.54 |  |  | .928 |  | .069 |  |
| Metric | 1130 | 2174.24 | 51.70 | .008 | .926 | .002 | .069 | .000 |
| Scalar | 1160 | 2243.93 | 69.69 | .001 | .923 | .003 | .069 | .000 |
| Strict | 1195 | 2327.34 | 83.41 | .001 | .920 | .003 | .070 | .001 |
| China (*n*_men_ = 183; *n*_women_ = 143) | | | | | | | | |
| Configural | 1096 | 1853.22 |  |  | .903 |  | .065 |  |
| Metric | 1126 | 1895.62 | 42.40 | .06 | .902 | .001 | .065 | .000 |
| Scalar | 1156 | 1988.79 | 93.17 | .001 | .894 | .008 | .066 | .001 |
| Strict | 1191 | 2100.79 | 111.99 | .001 | .884 | .010 | .068 | .002 |
| Sri Lanka (*n*_men_ = 183; *n*_women_ = 143) | | | | | | | | |
| Configural | 1098 | 1726.19 |  |  | .908 |  | .057 |  |
| Metric | 1128 | 1752.52 | 26.33 | .658 | .908 | .001 | .056 | .001 |
| Scalar | 1158 | 1806.09 | 53.57 | .005 | .905 | .003 | .057 | .001 |
| Strict | 1193 | 1857.55 | 51.46 | .036 | .902 | .003 | .056 | .001 |

*Note.* Changes in CFI less than |.01| and RMSEA less than |.015| are considered invariant.

**References**

Diedenhofen, B., & Musch, J. (2015). cocor: A Comprehensive Solution for the Statistical Comparison of Correlations. *PLOS ONE*, *10*(4), e0121945. https://doi.org/10.1371/journal.pone.0121945

Green, S. B., Akey, T. M., Fleming, K. K., Hershberger, S. L., & Marquis, J. G. (1997). Effect of the number of scale points on chi‐square fit indices in confirmatory factor analysis. *Structural Equation Modeling: A Multidisciplinary Journal*, *4*(2), 108–120. https://doi.org/10.1080/10705519709540064

Hu, L., & Bentler, P. M. (1999). Cutoff criteria for fit indexes in covariance structure analysis: Conventional criteria versus new alternatives. *Structural Equation Modeling: A Multidisciplinary Journal*, *6*(1), 1–55. https://doi.org/10.1080/10705519909540118

Statistics Solutions, 2022. Comparing correlation coefficients - Statistics Solutions. [online] Statistics Solutions. Available at: <https://www.statisticssolutions.com/comparing-correlation-coefficients/>.

Zou, G. Y. (2007). Toward using confidence intervals to compare correlations. *Psychological Methods*, *12*(4), 399–413. https://doi.org/10.1037/1082-989X.12.4.399
